# The effects of powdered drinks enriched with curcumin and probiotics on lipid profile and atherogenic indices in patients with metabolic syndrome: A randomized, double‐blinded, placebo‐controlled clinical trial

**DOI:** 10.1002/fsn3.3839

**Published:** 2023-11-20

**Authors:** Farzaneh Mohammadi, Mohammad Ali Mohsenpour, Zahra Sohrabi, Mehrdad Niakousari, Marjan Jeddi, Jafar Hassanzadeh, Gordon A. Ferns, Mohammad Hassan Eftekhari

**Affiliations:** ^1^ Department of Clinical Nutrition, School of Nutrition and Food Sciences Shiraz University of Medical Sciences Shiraz Iran; ^2^ Student Research Committee, School of Nutrition and Food Sciences Shiraz University of Medical Sciences Shiraz Iran; ^3^ Nutrition Research Center, School of Nutrition and Food Sciences Shiraz University of Medical Sciences Shiraz Iran; ^4^ Department of Food Science and Technology, College of Agriculture Shiraz University Shiraz Iran; ^5^ Endocrinology and Metabolism Research Center Shiraz University of Medical Sciences Shiraz Iran; ^6^ Department of Epidemiology, School of Health, Research Center for Health Sciences, Institute of Health Shiraz University of Medical Sciences Shiraz Iran; ^7^ Department of Medical Education Brighton & Sussex Medical School Brighton UK

**Keywords:** atherogenic, curcumin, lipid profile, metabolic syndrome, probiotics

## Abstract

Cardiovascular disease is prevalent globally and is the most common complication of metabolic syndrome (MetS). Previous studies have suggested that curcumin and probiotics may improve the lipid profile, so we aimed to investigate the effects of the edible powder enriched with these substances on lipid profile level and atherogenic indices such as Atherogenic Coefficient (AC), Castelli Risk Index‐I (CRI‐I), Castelli Risk Index‐II (CRI‐II), and Atherogenic Index of Plasma (AIP). In the present parallel randomized double‐blinded placebo‐controlled clinical trial, 124 people with MetS with overweight or obesity were randomly allocated to 4 groups and were followed up for 8 weeks. The participants received a low‐calorie diet and a daily sachet of enriched powder drink. The sachets contained either 10^9^ CFU of probiotics or 1 g of curcumin, or probiotic + curcumin (pro + cur), or placebo, respectively. The fasting lipid profile and atherogenic indices were measured at the beginning and end of the study. One hundred and fourteen participants completed the study. At the end of the study, the within‐ and between‐group comparisons showed no significant differences in lipid profile and atherogenic indices (*p* > .05). Based on the results of the current study, taking an oral powder containing 1 g curcumin and 10^9^ CFU probiotics for 8 weeks had no effect on the lipid profile level and atherogenic indices; however, more studies are recommended.

## Graphical abstract

The prevalence of metabolic syndrome and its complications such as cardiovascular diseases are increasing globally. Previous studies have suggested that curcumin and probiotics may improve the lipid profile. based on the result of the present study, taking an oral powdered drink containing 1 g curcumin and 109 CFU probiotics for 8 weeks had no effect on the lipid profile level and atherogenic indices in metabolic syndrome patients, however, more studies are recommended.

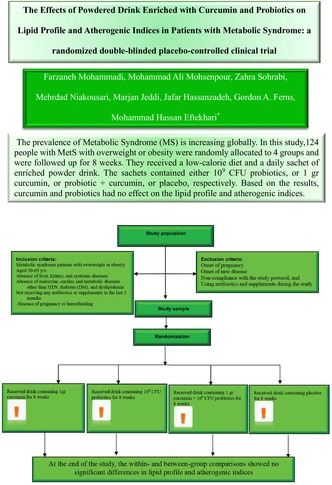

## 1 INTRODUCTION

Metabolic syndrome (MetS) is a clustering of cardiometabolic disorders that include abdominal obesity, impaired glucose tolerance, dyslipidemia, and hypertension (HTN). This syndrome is a public health challenge that increases with urban life, obesity, and sedentary lifestyles (McCracken et al., 2018; Safarian et al., 2019). About 14%–32% of the world's population and 30.4% of Iranians suffer from this syndrome (Farmanfarma et al., 2019). MetS is a chronic inflammatory condition triggered by the interaction of genetic and environmental factors. Insulin resistance (IR), visceral obesity, endothelial dysfunction, atherogenic dyslipidemia, genetic predisposition, increased blood pressure, increased coagulation conditions, and chronic stress contribute to the pathogenesis of MetS. Based on previous studies, a reduction of gut microbiota is associated with MetS and can increase inflammation, IR, and the risk of cardiovascular diseases (CVD) (Tenorio‐Jiménez et al., 2020).

CVD is one of the most common complications of MetS. Cardiometabolic indices such as the Atherogenic Index of Plasma (AIP), Castelli Risk Index‐I (CRI‐I), Castelli Risk Index‐II (CRI‐II), and Atherogenic Coefficient (AC) are exact and accessible tools that can predict and assess cardiometabolic risk. Several recent studies have used these indices to assess cardiometabolic risk. Total cholesterol (TC), triglyceride (TG), low‐density lipoprotein‐cholesterol (LDL‐C), and high‐density lipoprotein‐cholesterol (HDL‐C) are used to compute these indices (Sangouni et al., 2021).

Curcumin is a polyphenolic substance found in turmeric rhizomes (*Curcuma longa* L.) and is a yellow‐orange pigment with antioxidant, anti‐inflammatory, anti‐thrombosis, anti‐sclerosis, and heart protection properties (Agrawal & Mishra, 2010; Ghazimoradi et al., 2017; Shen & Ji, 2012). Turmeric is a common spice in Middle Eastern and Southeast Asian cooking and is extensively used in herbal and traditional medicine fields (Mohammadi et al., 2019). Curcumin and its metabolites revealed positive biological effects on the gut microbiota. Moreover, the gut microbiota raised the bioavailability of curcumin (Scazzocchio et al., 2020). Although the effects of curcumin on lipid profile are controversial, in several studies, curcumin improved the lipid profile levels in patients with MetS and a high risk of CVD (Qin et al., 2017; Yang et al., 2014), and in some studies, it did not affect lipid profile and atherogenic indices (Baum et al., 2007; Naseri et al., 2022; Saberi‐Karimian et al., 2018).

Probiotics are reported to improve the risk factors associated with MetS and CVD by preventing the instability of the gut microbiota, which is called dysbiosis, and balancing the intestinal flora. Some clinical trials have reported that treatment with probiotics has protective effects on lipid profile levels and atherogenic indices (Gadelha & Bezerra, 2019; Miglioranza Scavuzzi et al., 2015; Yadav et al., 2019), while others did not show any significant effect (Sabico et al., 2017; Stadlbauer et al., 2015).

Since CVD is one of the most common complications in patients with MetS and cardiometabolic indices such as AIP, Castelli risk indices, and AC are valid and accessible indices for predicting cardiometabolic risk, in addition to the lack of studies that have investigated the combined effects of curcumin and probiotics on these indicators, the present study examined the outcomes of powder enriched with the two mentioned ingredients on lipid profile levels and atherogenic indices.

## 2 MATERIALS AND METHODS

### 2.1 Study design

The present parallel randomized double‐blinded placebo‐controlled clinical trial was performed according to the declaration of Helsinki and good clinical practice guidelines at Imam Reza Clinic and Laboratory of the School of Nutrition and Food Sciences, Shiraz University of Medical Sciences, Shiraz, Iran. The protocol of the study was approved by the Ethics Committee of Shiraz University of Medical Sciences, Shiraz, Iran (IR.SUMS.SCHEANUT.REC.1401.099). Also, the study was registered on the Iranian Registry of Clinical Trials (IRCT20220315054290N2).

### 2.2 Participants

For this study, the endocrinologist screened patients for eligibility. Inclusion criteria included patients aged 30–65 years and diagnosed with MetS based on the National Cholesterol Education Program Adult Treatment Panel III (NCEP ATP III) criteria (Pasternak, 2003) with overweight (25 < BMI <29.9) or obesity (BMI ≥30 kg/m^2^), absence of liver, kidney, and systemic diseases, absence of endocrine, cardiac, and metabolic diseases other than HTN, diabetes (DM), and dyslipidemia, not receiving any antibiotics or supplements in the last 3 months, and absence of pregnancy or breastfeeding. Participants who have 3 or more of the following criteria are defined as having MetS according to the ATP III criteria: waist circumference (WC) > 88 cm for women and > 102 cm for men; TG ≥150 mg/dL; HDL‐C < 50 mg/dL for women and < 40 mg/dL for men; fasting blood sugar (FBS) ≥ 110 mg/dL; systolic blood pressure (SBP) ≥ 130 mmHg; or diastolic blood pressure (DBP) ≥ 85 mmHg. Exclusion criteria included the onset of pregnancy or a new disease, non‐compliance with the study protocol, and using antibiotics and supplements during the study period. All of the participants were informed about the study protocol before the study. Also, they signed the informed consent.

### 2.3 Sample size

According to the previous study (Ahmadian et al., 2022) and considering the reduction of 0.15 in the AIP index, standard deviation (SD) = 0.2, a significance level of 0.05, and a power of 80%, the sample size was calculated as 28 people in each group, and after taking into account a possible 10% attrition, 31 participants per group were required.

### 2.4 Randomization

A person rather than the main investigator divided the participants into 4 groups (3 intervention and 1 control group) randomly and equally by a computer method using balanced block randomization with a block size of 4 (1:1:1:1 ratio). Moreover, the third party determined the random allocation sequences and categorized them in opaque envelopes named A, B, C, and D to conceal the allocation. Each participant enrolled in the study based on the opened envelope containing the assigned group.

### 2.5 Interventions

To homogenize the nutritional behaviors and prevent their confounding effects, the 4 groups completed a 2‐week run‐in period along with following the lifestyle amendment recommendations before the study. Afterward, the participants were assigned to receive one sachet per day of enriched powder along with a low‐calorie diet for 8 weeks. Sachets were filled with either 10^9^ CFU probiotics (Lactobacillus acidophilus and Lactobacillus rhamnosus strains) (Parsi Lact, Pardis Roshd Mehregan Co., Shiraz, Iran), or 1 gr of curcumin (Karen Inc., Tehran, Iran), or 1 gr of curcumin +10^9^ CFU probiotics (cur + pro), respectively, or a placebo powder. The participants were asked to dissolve the powder in water and drink it. The dose of curcumin and probiotic was determined according to the previous study (Panahi et al., 2014; Rerksuppaphol & Rerksuppaphol, 2015), and the preparation of powders was explained in the previous research that has not been published yet (Mohsenpour et al., 2023). The low‐calorie diet included a reduction of 500 kcal/day of Total Energy Expenditure (TEE), 50%–55% carbohydrates, 15%–20% protein, and 25%–30% fat. TEE was estimated using the Resting Energy Expenditure (REE) (Mifflin‐St Jeor formula) and physical activity coefficient (Mahan & Raymond, 2016).

### 2.6 Blinding

The appearance of the sachets was similar. In addition, the powders’ color, smell, and taste were identical. All powders had an orange taste and color. The sachets were labeled A, B, C, and D to be blinded. Also, the participants and the researcher were blinded by the interventions and group allocation.

### 2.7 Demographic assessments

A questionnaire that comprised information about gender, age, education, marital status, job, income, diseases, smoking, and alcohol consumption was used for demographic assessments at the beginning of the study.

### 2.8 Blood pressure assessment

Patients were asked to rest 5 min before BP measurements in a sitting position. BP was measured by an expert person using a sphygmomanometer (Riester Precisa‐N, Germany) from their right hand at the beginning of the study. The right hand of the participant was horizontal, stretched, and level with the heart.

### 2.9 Anthropometric assessments

Height was measured to the nearest 0.1 cm through a stadiometer (Seca®, Germany) without shoes, scarf, and hat, while the shoulders, buttocks, and heels were attached to the wall and the head was in the Frankfurt situation. Weight and body mass index (BMI) were measured with the least clothing and without shoes and socks by a bioelectrical impedance analysis (BIA) device (Tanita® BC‐418, Japan). Waist circumference (WC) was measured with an accuracy of 0.1 cm by an inelastic tape measure at the narrowest point between the last rib and the iliac crest without clothing. The tape was parallel to the ground, and the participants were in a standing position.

### 2.10 Biochemical assessments

Five ml of venous blood was taken from each participant after 8–10 h of overnight fasting at the beginning and end of the study phase. Sera were separated by centrifugation (3000 *g* for 5 min) and stored at −80°C until the final analyses. The standard enzymatic method was used for measuring FBG, TC, HDL‐C, LDL‐C, and TG using automated colorimetric methods (auto‐analyzer BT‐1500, Italy) and commercial kits (Biorex Inc., Iran). FBG was measured just at the beginning of the study, but the lipid profile was measured both at the beginning and the end of the study.

### 2.11 Atherogenic indices assessments

Atherogenic indices (AC, AIP, CRI‐I, and CRI‐II) were computed by the following equations at the beginning and end of the study:

$$\mathrm{AC}=\left(\mathrm{TC}-\mathrm{HDL}-C\right)/\mathrm{HDL}–C$$

$$\mathrm{AIP}=\log\;\left(\mathrm{TG}/\mathrm{HDL}-C\right)$$

CRI‐I=TC/HDL–C

CRI‐II=LDL−C/HDL–C

### 2.12 Dietary intake assessment

Participants' dietary intakes were assessed by a 3‐day food recall (2 regular days and a weekend) before and after the intervention. Then these food records were changed to grams according to the usual household scales of Iranian food and evaluated by Nutritionist 4 software (First Databank Inc., San Bruno, CA, USA) modified for Iranian foods. Amounts of calories, carbohydrates, protein, fat, cholesterol, saturated fatty acid (SFA), polyunsaturated fatty acid (PUFA), monounsaturated fatty acid (MUFA), zinc, selenium, copper, vitamins C and E, and fiber were calculated.

### 2.13 Physical activity assessment

The International Physical Activity Questionnaire Short Form (IPAQ‐SF) with seven questions was applied to assess physical activity before and after the study. These questions are about physical activity's intensity (light, moderate, and vigorous) and duration (minutes). The metabolic equivalent (MET) of light, medium, and vigorous physical activities is 3.3, 4, and 8, respectively. The quantity of physical activity of each participant is calculated by multiplying the intensity (MET) by the duration (minutes) of physical activity (MET·min/week).

### 2.14 Compliance assessment

We called the participants every week to remind them to use the powders. The participants also documented the powder eaten every day on a checklist. After 4 weeks, they were asked to refer to the clinic to receive the next week's sachets and return the uneaten powders. Individuals who ate less than 80% of the sachets were regarded as non‐adherent and excluded from the final evaluation. Also, in the case of diet, individuals whose compliance rate was less than 50% were removed from the analysis.

### 2.15 Statistical analysis

Statistical Package for Social Sciences (SPSS) software (version 20.0, SPSS Inc., Chicago, IL, USA) and the per‐protocol method were used to perform data analysis. The significance level was considered less than 0.05. The Chi‐square test was used for the between‐group comparison of the categorical variables. Within‐group comparisons of variables were accomplished by a paired *t*‐test. To compare variables between groups, first the mean difference (after–before) was calculated, then the mean difference in four groups was compared using the analysis of variance (ANOVA) test. The effect of covariates was adjusted by an analysis of covariance (ANCOVA).

## 3 RESULTS

**FIGURE 1:**
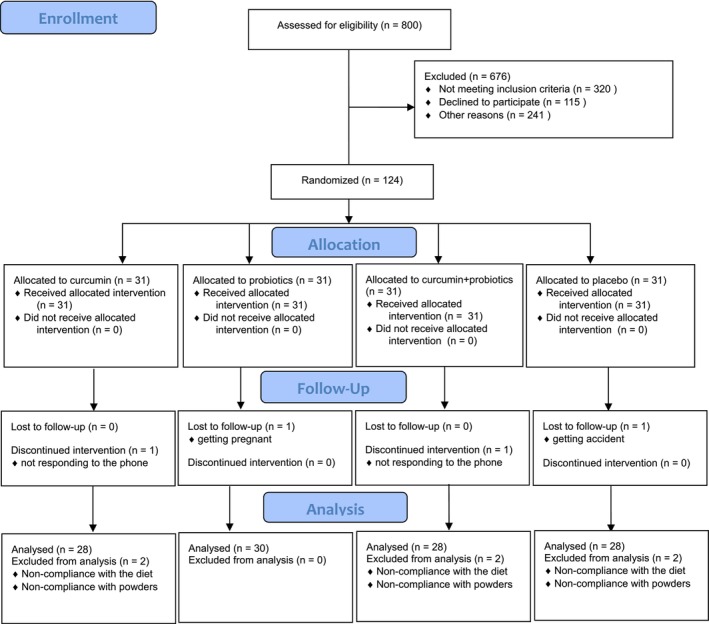
CONSORT flow diagram.

Out of 800 people assessed for eligibility, 124 (102/22, female/male) were eligible to attend the study. These participants were randomly allocated to one of 4 groups of 31 with a ratio of 1:1:1:1 and were recruited and followed up from June to September 2022. Participants were excluded from each group as follows: three participants in the curcumin group due to not answering the phone (*n* = 1), non‐compliance with the diet (*n* = 1), and non‐compliance with eating powders (*n* = 1), 3 in the placebo group because of an accident (*n* = 1), non‐compliance with eating powders (*n* = 1), and non‐compliance with the diet (*n* = 1), 3 in the cur + pro group due to not answering the phone (*n* = 1), non‐compliance with the diet (*n* = 1), and non‐compliance with eating powders (*n* = 1), and 1 in the probiotics group because of pregnancy (*n* = 1) (Figure 1). No adverse effects of powder use were observed in the participants.

According to Tables 1 and 2, no significant difference was observed between the study groups in terms of demographic information and baseline values of FBG, SBP, DBP, and anthropometric variables (*p* > .05) except for BMI (*p* = .004) and WC (*p* = .002). Also, there was no significant difference in the changes in food intake and physical activity between the study groups (*p* > .05) (Table 3). After adjustment for baseline BMI and WC, within‐ and between‐group comparisons of lipid profile level and atherogenic indices did not indicate any significant difference (*p* > .05) (Table 4).

**TABLE 1:** Demographic characteristics of the participants (*n* = 114).

Variables	Cur (*n* = 28)	Pro (*n* = 30)	Cur + pro (*n* = 28)	Placebo (*n* = 28)	*p*‐Value^a^
Age (year) Mean ± SE	49.39 ± 1.31	49.50 ± 1.13	47.39 ± 1.67	48.96 ± 1.28	.675
Gender, *n* (%)					
Female	24 (85.7)	27 (90)	21 (75)	24 (85.7)	.451
Male	4 (14.3)	3 (10)	7 (25)	4 (14.3)	
Marital status, *n* (%)					
Single	4 (14.3)	3 (10)	3 (10)	2 (7.1)	.857
Married	24 (85.7)	27 (90)	25 (89.3)	26 (92.9)	
Education status, *n* (%)					
Illiterate & elementary	12 (42.9)	16 (53.3)	11 (39.3)	11 (39.3)	.664
Diploma & upper diploma	16 (57.1)	14 (46.7)	17 (60.7)	17 (60.7)	
Job‐status, *n* (%)					
Employed	4 (14.3)	4 (13.3)	10 (35.7)	5 (17.9)	.124
Unemployed	24 (85.7)	26 (86.7)	18 (64.3)	23 (82.1)	
Income status, *n* (%)					
Under 120 $	15 (53.6)	24 (80)	16 (57.1)	17 (60.7)	.156
120 $ or higher	13 (46.4)	6 (20)	12 (42.9)	11 (39.3)	
Current smoking, *n* (%)					
Yes	3 (10.7)	8 (26.7)	8 (28.6)	9 (32.1)	.253
Alcohol drinking, *n* (%)					
Yes	0 (0)	0 (0)	0 (0)	0 (0)	1
Diabetes disease, *n* (%)					
Yes	22 (78.6)	28 (93.3)	25 (89.3)	22 (78.6)	.276
No	6 (21.4)	2 (6.7)	3 (10.7)	6 (21.4)	
Hypertension disease, *n* (%)					
Yes	11 (39.3)	14 (46.7)	14 (50)	16 (57.1)	.604
No	17 (60.7)	16 (53.3)	14 (50)	12 (42.9)	
Dyslipidemia disease, *n* (%)					
Yes	19 (67.9)	23 (76.7)	20 (71.4)	22 (78.6)	.790
No	9 (32.1)	7 (23.3)	8 (28.6)	6 (21.4)	

*Note*: A *p*‐value <.05 is considered significant.

Abbreviations: Cur, curcumin; Cur + Pro: Curcumin + Probiotics; Pro, Probiotics.

^a^ *p*‐Value for age is computed using ANOVA test, while chi‐square test was used for others.

**TABLE 2:** Baseline fasting blood sugar, blood pressure, and anthropometric variables of the participants (*n* = 114).

Variables	Cur (*n* = 28)	Pro (*n* = 30)	Cur + pro (*n* = 28)	Placebo (*n* = 28)	*p*‐Value^a^
FBS (mg/dL)	136.10 ± 10.72	145.73 ± 8.33	167.60 ± 13.34	137.75 ± 10.88	.163
SBP (mmHg)	130.78 ± 2.81	129.96 ± 2.81	130.25 ± 3.10	134.64 ± 3.40	.675
DBP (mmHg)	81.32 ± 2.36	80.63 ± 1.87	79.64 ± 1.91	82.75 ± 2.52	.787
Height (cm)	161.14 ± 1.69	159.63 ± 1.54	162.07 ± 1.63	159.14 ± 1.61	.561
Weight (kg)	85.11 ± 1.96	84.36 ± 2.29	77.10 ± 2.15	80.54 ± 2.99	.072
BMI (kg/m^2^)	32.83 ± 0.71	33.18 ± 0.84	29.36 ± 0.76	31.65 ± 0.85	.004
WC (cm)	110.28 ± 1.54	110.60 ± 1.37	102.92 ± 1.81	106.53 ± 1.59	.002

Abbreviations: BMI, body mass index; Cur + Pro, Curcumin + Probiotics; Cur, curcumin; DBP, diastolic blood pressure; FBS, fasting blood sugar; Pro, Probiotics; SBP, systolic blood pressure; WC, waist circumference.

*Note*: Data reported as Mean ± Standard Error (SE). A *p*‐value <.05 is considered significant.

^a^ Between‐group comparison (ANOVA test).

**TABLE 3:** Changes (after – before) in dietary intake and physical activity of the participants (*n* = 114) at the end of the study.

Variables	Cur (*n* = 28)	Pro (*n* = 30)	Cur + Pro (*n* = 28)	Placebo (*n* = 28)	*p*‐Value^a^
Energy (kcal/day)	−505.10 ± 69.02	−438.98 ± 51.93	−544.03 ± 449.22	−623.06 ± 124.36	.814
Carbohydrate (g/day)	−75.29 ± 14.14	− 73.22 ± 10.00	−81.07 ± 15.07	−90.30 ± 18.44	.275
Protein (g/day)	−15.18 ± 19.10	−18.73 ± 3.80	−15.98 ± 3.06	−21.57 ± 5.12	.526
Fat (g/day)	−15.67 ± 4.72	−8.90 ± 2.96	−16.18 ± 5.14	−20.67 ± 6.29	1.01
Fiber (g/day)	−1.74 ± 0.78	−1.33 ± 0.71	−0.91 ± 0.43	−1.70 ± 0.55	.786
Chol (mg/day)	−12.52 ± 30.65	−69.30 ± 29.17	−63.87 ± 26.14	−80.46 ± 40.13	.453
SFA (g/day)	−2.25 ± 1.53	−0.69 ± 0.90	−9.39 ± 8.24	−3.59 ± 1.46	.489
PUFA (g/day)	−1.17 ± 2.02	−2.55 ± 1.79	−61.44 ± 58.35	−8.54 ± 3.32	.397
MUFA (g/day)	−1.45 ± 1.64	−0.76 ± 0.89	−12.16 ± 9.87	−5.53 ± 1.85	.359
Zinc (mg/day)	−0.94 ± 0.68	−1.42 ± 0.45	−9.90 ± 6.04	−2.22 ± 0.79	.129
Copper (mg/day)	−0.47 ± 0.16	−0.52 ± 0.21	−0.45 ± 0.11	−0.99 ± 0.30	.226
Selenium (mg/day)	−.00 ± 0.00	−0.01 ± 0.00	−0.01 ± 0.00	−0.01 ± 0.00	.693
Vitamin C (mg/day)	−21.96 ± 9.16	−21.97 ± 20.58	−29.16 ± 16.55	−23.05 ± 11.20	.985
Vitamin E (mg/day)	−0.82 ± 1.16	−2.22 ± 1.05	−29.84 ± 29.99	−3.79 ± 1.68	.461
Physical activity (MET min/week)	112.85 ± 41.81	107.65 ± 36.80	24.10 ± 48.43	133.91 ± 43.91	.292

*Note*: Data reported as Mean ± SE. A *p*‐value <.05 is considered significant.

Abbreviations: Chol, cholesterol; Cur + Pro, Curcumin + Probiotics; Cur, curcumin; MUFA, monounsaturated fatty acid; Pro, Probiotics; PUFA, polyunsaturated fatty acid; SFA, saturated fatty acid.

^a^ Between‐group comparison (ANOVA test).

**TABLE 4:** Changes (after–before) in lipid profile level and atherogenic indices of the participants (*n* = 114) at the end of the study.

Variables	Cur (*n* = 28)	Pro (*n* = 30)	Cur + Pro (*n* = 28)	Placebo (*n* = 28)	*p*‐Value^a^
TC (mg/dL)					
Before	170.17 ± 7.39	173.56 ± 7.17	175.00 ± 7.63	179.71 ± 7.32	.836
After	173.26 ± 7.61	178.42 ± 7.39	184.77 ± 7.85	171.36 ± 7.54	.611
Change	3.09 ± 5.31	4.86 ± 5.15	9.76 ± 5.48	−8.35 ± 5.26	.102
*p*‐Value^b^	.607	.347	.081	.055	
TG (mg/dL)					
Before	189.26 ± 21.08	168.07 ± 20.47	217.85 ± 21.76	166.56 ± 20.89	.297
After	193.71 ± 25.86	181.17 ± 25.12	236.12 ± 26.70	150.97 ± 25.63	.146
Change	4.45 ± 14.42	13.09 ± 14.00	18.24 ± 14.88	−15.59 ± 14.29	.356
*p*‐Value^b^	.921	.496	.184	.139	
LDL‐C (mg/dL)					
Before	140.91 ± 9.58	149.71 ± 9.30	148.90 ± 9.89	156.48 ± 9.49	.721
After	145.25 ± 10.06	157.05 ± 9.77	157.14 ± 10.38	145.72 ± 9.97	.712
Change	4.33 ± 6.47	7.34 ± 6.28	8.24 ± 6.67	−10.76 ± 6.14	.130
*p*‐Value^b^	.578	.233	.208	.051	
HDL‐C (mg/dL)					
Before	42.08 ± 1.48	40.94 ± 1.43	40.91 ± 1.52	42.24 ± 1.46	.867
After	42.53 ± 1.83	40.16 ± 1.49	41.78 ± 1.77	41.64 ± 1.64	.786
Change	0.66 ± 1.11	−0.55 ± 1.08	0.53 ± 1.14	−0.72 ± 1.10	.731
*p*‐Value^b^	.460	.622	.794	.564	
AC					
Before	3.10 ± 0.18	3.34 ± 0.17	3.34 ± 0.19	3.34 ± 0.18	.742
After	3.11 ± 0.20	3.53 ± 0.20	3.64 ± 0.21	3.26 ± 0.20	.255
Change	−0.00 ± 0.11	0.18 ± 0.11	0.30 ± 0.12	−0.07 ± 0.11	.101
*p*‐Value^b^	.957	.063	.047	.461	
AIP					
Before	0.56 ± 0.04	0.58 ± 0.04	0.64 ± 0.05	0.56 ± 0.04	.716
After	0.56 ± 0.05	0.62 ± 0.05	0.64 ± 0.05	0.53 ± 0.05	.491
Change	−0.03 ± 0.03	0.04 ± 0.03	0.00 ± 0.03	−0.02 ± 0.03	.446
*p*‐Value^b^	.387	.281	.573	.473	
CRI‐I					
Before	4.10 ± 0.18	4.34 ± 0.17	4.34 ± 0.19	4.34 ± 0.18	.742
After	4.11 ± 0.20	4.53 ± 0.20	4.64 ± 0.21	4.26 ± 0.20	.255
Change	0.00 ± o.11	0.18 ± 0.11	0.30 ± 0.12	−0.07 ± 0.11	.101
*p*‐Value^b^	.957	.063	.047	.461	
CRI‐II					
Before	3.38 ± 0.22	3.78 ± 0.21	3.65 ± 0.22	3.78 ± 0.21	.524
After	3.41 ± 0.24	4.00 ± 0.23	3.92 ± 0.24	3.65 ± 0.23	.284
Change	0.03 ± 0.13	0.22 ± 0.13	0.27 ± 0.14	−0.13 ± 0.13	.143
*p*‐Value^b^	.906	.074	.072	.319	

*Note*: Data reported as Mean ± SE. A *p*‐value <.05 is considered significant.

Abbreviations: AC, atherogenic coefficient; AIP, atherogenic index of plasma; CRI‐I, castelli risk index I; CRI‐II, castelli risk index II; Cur + Pro, Curcumin + Probiotics; Cur, curcumin; HDL‐C, high‐density lipoprotein cholesterol; LDL‐C, low‐density lipoprotein cholesterol; Pro, Probiotics; TC, total cholesterol; TG, triglyceride.

^a^ Between‐group comparison (ANCOVA test adjusted by BMI and WC).

^b^ Within‐group comparison (Paired *t*‐test).

## 4 DISCUSSION

To the best of our knowledge, the present study was the first clinical trial that investigated the effect of the co‐administration of curcumin and probiotics on lipid profiles and atherogenic indices. The results demonstrated that consuming 1g of curcumin and 10^9^ CFU of probiotics alone or in combination for 8 weeks did not affect lipid profile and atherogenic indices. Although the amounts of lipid profile and atherogenic indices increased in the intervention groups and decreased in the placebo group, these changes were not clinically and statistically significant and may be due to the greater compliance of the placebo group with the diet and more changes in caloric intake and physical activity in this group.

### 4.1 Effects of curcumin on lipid profiles and atherogenic indices

Dyslipidemia is one of the indicators of MetS (McCracken et al., 2018). The effects of curcumin, which is the active ingredient of turmeric, on the lipid profile and atherogenic indicators have been investigated (Baum et al., 2007; Qin et al., 2017; Saberi‐Karimian et al., 2018; Yang et al., 2014). Atherogenic indices are novel indicators for monitoring CVDs (Naseri et al., 2022).

In line with the present study, in some previous studies, supplementation with curcumin in MetS and other diseases did not have an improving effect on lipid profile level and atherogenic indices (Adibian et al., 2019; Baum et al., 2007; Naseri et al., 2022; Saberi‐Karimian et al., 2018; Sohaei et al., 2019). Nonetheless, in some animal and human studies, supplementation with curcumin led to improvements in lipid profiles and some atherogenic indicators (Ghelani et al., 2019; Qin et al., 2017; Yang et al., 2014). Also, in a recently published study, curcumin extract in carrot and orange mixed drinks reduced triglyceride levels in hemodialysis patients but had no effect on other lipid profile parameters (Alvarenga et al., 2023). Curcumin is thought to improve the lipid profile through different mechanisms, as follows. Curcumin reduces serum cholesterol levels by preventing the absorption of cholesterol (Arafa, 2005). Molecular and cellular mechanisms for lipid regulation by curcuminoids include inhibition of de novo lipid biosynthesis through regulation of main lipogenic elements such as 3‐hydroxy‐3‐methylglutaryl CoA (HMG‐CoA) reductase, fatty acid synthase, and sterol regulatory element‐binding protein‐1/2 (SREBP‐1/2). Also, curcuminoids stimulate the catabolism and biliary excretion of lipids and the release of fat from adipose tissue (Panahi et al., 2014). Curcumin also reduces serum TG levels by reducing the activity of the lipoprotein lipase enzyme (Aggarwal & Harikumar, 2009). On the other hand, curcumin improves the lipid profile by stimulating the beta‐oxidation of fatty acids and hindering fatty acid synthesis (Sukandar et al., 2010).

In spite of these mechanisms, the dissimilarities in results may be due to the difference in the absorption of curcumin by people (Baum et al., 2007), the low bioavailability of curcumin (Panahi et al., 2014), the instability of curcumin in the intestinal pH (Anand et al., 2007), and the consumption of curcumin in the fasting state (Zingg et al., 2013), as well as the low dose, short duration of the study, and small sample size.

### 4.2 Effects of probiotics on lipid profiles and atherogenic indices

Recently, it has been found that MetS is associated with instability in the gut microbiota. Probiotics are living microorganisms that improve intestinal flora and thereby affect components of MetS such as dyslipidemia (Tenorio‐Jiménez et al., 2020).

Although similar to the current study, in some studies, probiotics consumption had no effect on lipid profile (Sabico et al., 2017; Stadlbauer et al., 2015), some studies reported positive results of probiotics intake in this field (Miglioranza Scavuzzi et al., 2015; Yadav et al., 2019). According to our knowledge, previous studies have rarely calculated atherogenic indices. In Ahmadian et al.'s (2022) study, probiotic supplementation in T2DM patients did not affect CRI‐I. However, in Ahmadian et al.'s (2022) study on probiotic supplement consumption and in Ejtahed et al.'s (2011) study on probiotic‐enriched yogurt consumption, a significant decrease in the AIP index was observed (Ejtahed et al., 2011). The proposed mechanisms for the effects of probiotics on lipid profiles are as follows. First, probiotics bind cholesterol to their cell walls and prevent it from being absorbed by the digestive system into the bloodstream. Second, by generating short‐chain fatty acids such as propionate, probiotics suppress MHG‐CoA reductase, which plays a role in cholesterol synthesis. Third, probiotics contain bile salt hydrolase, which inhibits the absorption of cholesterol in the enterohepatic cycle by deconjugating bile acids. Therefore, reducing the absorption of cholesterol in this cycle leads to the synthesis of more bile acids that use circulating cholesterol (Hara et al., 1999; Noh & Gilliland, 1993; Zhuang et al., 2012). Fourth, probiotics decrease TG and thereby increase HDL‐C. In this way, in the hypertriglyceridemic state, HDL particles become filled with TG through the exchange of cholesterol with TG. These TG‐rich HDL particles are metabolized faster than normal HDL particles in the liver; as a result, TG decreases and HDL increases (Eslamparast et al., 2014).

These differences in results could be due to differences in probiotic strains, probiotic dose received, study duration, and carriers used for probiotic administration (milk, yogurt, cheese, or capsules).

### 4.3 Effects of curcumin + probiotics on lipid profiles

There have been no previous human studies on the synergistic effect of curcumin and probiotics on lipid profiles and atherogenic indices. However, in an animal study, receiving curcumin and the probiotic *Lactobacillus acidophilus* alone or together decreased TG and TC in rats with MetS (Kapar & Ciftci, 2020). In another study on diabetic rats, TC in the turmeric group and LDL‐C in both the turmeric group and the turmeric + probiotic group decreased. Moreover, HDL‐C increased in all three groups (Maha, 2013). The effects of curcumin and probiotics alone on lipid profiles were discussed in the previous sections. Also, the synergistic effects of probiotics and curcumin can be due to the mutual relationship between curcumin, probiotics, and gut microbiota (Scazzocchio et al., 2020).

### 4.4 Strengths and limitations

The investigation of the combination group containing cur + pro at the same time for the first time was the main strength. Also, the use of an orange flavor that made the powder edible and palatable to the participants and increased their compliance was another strength of this study. Limitations of the study included not measuring changes in the gut microbiota due to a low budget, not using different doses of curcumin and probiotics, as well as the small sample size and short duration of the follow‐up. Thus, the results of the present study cannot be generalized to MetS patients.

## 5 CONCLUSION

Based on the results of the present study, receiving oral powder enriched with curcumin and probiotics did not affect the fasted lipid profile and atherogenic indicators of patients with MetS. However, further studies with various doses or durations are warranted to better elucidate the exact effects or mechanisms of the actions of curcumin and probiotics and their combination.

## AUTHOR CONTRIBUTIONS

**Farzaneh Mohammadi:** Conceptualization (equal); data curation (equal); formal analysis (equal); investigation (equal); methodology (equal); project administration (equal); writing – original draft (equal); writing – review and editing (equal). **Mohammad Ali Mohsenpour:** Data curation (equal); investigation (equal); methodology (equal); project administration (equal); writing – review and editing (equal). **Zahra Sohrabi:** Investigation (equal); methodology (equal); project administration (equal); writing – review and editing (equal). **Mehrdad Niakousari:** Conceptualization (equal); investigation (equal); methodology (equal); project administration (equal); writing – review and editing (equal). **Marjan Jeddi:** Investigation (equal); methodology (equal); project administration (equal); writing – review and editing (equal). **Jafar Hassanzadeh:** Formal analysis (equal); investigation (equal); methodology (equal); writing – review and editing (equal). **Gordon A. Ferns:** Investigation (equal); methodology (equal); project administration (equal); writing – review and editing (equal). **Mohammad Hassan Eftekhari:** Conceptualization (equal); methodology (equal); project administration (equal); supervision (lead); writing – review and editing (equal).

## FUNDING INFORMATION

This study was financially supported by the Vice‐chancellory of Research and Technology, Shiraz University of Medical Sciences, Shiraz, Iran (grant number: SG‐01‐74).

## CONFLICT OF INTEREST STATEMENT

The authors declare no conflict of interest.

## Data Availability

The data that support the findings of this study are available on request from the corresponding author. The data are not publicly available due to privacy or ethical restrictions.
